# Syndecans Reside in Sphingomyelin-Enriched Low-Density Fractions of the Plasma Membrane Isolated from a Parathyroid Cell Line

**DOI:** 10.1371/journal.pone.0032351

**Published:** 2012-03-01

**Authors:** Katarzyna A. Podyma-Inoue, Miki Hara-Yokoyama, Tamayuki Shinomura, Tomoko Kimura, Masaki Yanagishita

**Affiliations:** 1 Section of Biochemistry, Graduate School of Medical and Dental Sciences, Tokyo Medical and Dental University, Tokyo, Japan; 2 Section of Hard Tissue Engineering, Department of Hard Tissue Engineering, Graduate School of Medical and Dental Sciences, Tokyo Medical and Dental University, Tokyo, Japan; Consejo Superior de Investigaciones Cientificas, Spain

## Abstract

**Background:**

Heparan sulfate proteoglycans (HSPGs) are one of the basic constituents of plasma membranes. Specific molecular interactions between HSPGs and a number of extracellular ligands have been reported. Mechanisms involved in controlling the localization and abundance of HSPG on specific domains on the cell surface, such as membrane rafts, could play important regulatory roles in signal transduction.

**Methodology/Principal Findings:**

Using metabolic radiolabeling and sucrose-density gradient ultracentrifugation techniques, we identified [^35^S]sulfate-labeled macromolecules associated with detergent-resistant membranes (DRMs) isolated from a rat parathyroid cell line. DRM fractions showed high specific radioactivity ([^35^S]sulfate/mg protein), implying the specific recruitment of HSPGs to the membrane rafts. Identity of DRM-associated [^35^S]sulfate-labeled molecules as HSPGs was confirmed by Western blotting with antibodies that recognize heparan sulfate (HS)-derived epitope. Analyses of core proteins by SDS-PAGE revealed bands with an apparent MW of syndecan-4 (30–33 kDa) and syndecan-1 (70 kDa) suggesting the presence of rafts with various HSPG species. DRM fractions enriched with HSPGs were characterized by high sphingomyelin content and found to only partially overlap with the fractions enriched in ganglioside GM1. HSPGs could be also detected in DRMs even after prior treatment of cells with heparitinase.

**Conclusions/Significance:**

Both syndecan-1 and syndecan-4 have been found to specifically associate with membrane rafts and their association seemed independent of intact HS chains. Membrane rafts in which HSPGs reside were also enriched with sphingomyelin, suggesting their possible involvement in FGF signaling. Further studies, involving proteomic characterization of membrane domains containing HSPGs might improve our knowledge on the nature of HSPG-ligand interactions and their role in different signaling platforms.

## Introduction

Heparan sulfate proteoglycans (HSPGs) are ubiquitous molecules among animal cells and are one of the basic constituents of plasma membranes. HSPGs are glycoproteins in which the protein core is substituted with heparan sulfate chains; specific patterns of sulfation endow HSPGs with their unique biological functions. For instance, HSPGs show specific molecular interactions with a number of heparin-binding growth factors, cytokines, plasma membrane proteins (involved in cell-cell or cell-extracellular matrix interactions), and pathogens (such as viruses and plasmodium) [1], [2]. HSPGs serve as co-factor/co-receptors for the cellular prion proteins [3], [4]. HSPGs intercalated into cell membrane through their core proteins (e.g., syndecan family) or linked by glycosylphosphatidylinositol (GPI)-anchor (e.g., glypican family) bind to a number of extracellular ligands, intercept and regulate biological signals coming into the cells. The biological functions of HSPGs are regulated by several mechanisms, including those involved in HSPGs' expression (with proper carbohydrate modification), their targeting and maintenance on the cell surface, their shedding from the cell surface, and finally their endocytosis and intracellular degradation. In addition, the localization of HSPGs to specific domains on the cell surface likely plays an important regulatory role.

Syndecans are one of the major HSPGs present on the cell surface. They are type I transmembrane proteins with variable extracellular domain bearing glycosaminoglycan attachment sites and a well-conserved short cytoplasmic domain. Four members found in mammalian cells carry various combinations of heparan sulfate (HS) and chondroitin sulfate chains. Temporal and spatial patterns of expression of each syndecan family are tightly regulated throughout the development and are often associated with cell differentiation, morphology or organogenesis [1]. Syndecans modulate the activity of cell surface-bound ligands by presenting them in the active conformation to their receptors and by taking them up into endosome/lysosomal compartments. In spite of a high degree of structural conservation, syndecans differ in their generation of intracellular signals suggesting an important role for their localization on the specific areas of the cell surface and pericellular milieu [5].

Plasma membranes of mammalian cells are composed of distinct domains, such as membrane rafts. Membrane rafts are enriched with cholesterol, glycosphingolipids, GPI-anchored proteins and signaling molecules. They have been implicated in signal transduction, membrane trafficking and lipid sorting [6], [7]. Membrane rafts are dynamic structures believed to be formed during signaling or undergo structural changes by recruiting/excluding specific lipids or proteins during signal transduction [8], [9]. However, the actual physical properties and turnover of such membrane domains are not fully characterized. For instance, one important issue is the compositional and functional heterogeneity of rafts, related to the diversity of lipid and protein components associated with the membrane raft structures. Recent immunoelectron microscopic studies using Jurkat T cells have shown different cell surface distribution of GM1 ganglioside-rich and sphingomyelin (SM)-rich domains [10], [11]. GM1-rich domains mediate T cell receptor activation [12], while SM-rich domains mainly participate in G protein-coupled receptor-dependent signaling, although mobilization of calcium ions and activation of ERK1/2 have been also reported [10]. The diversity in lipid composition may partially influence diversity of protein composition, since certain transmembrane proteins, e.g., influenza virus hemagglutinin or linker for activation of T cells, are localized to the membrane rafts due to protein-lipid or protein-protein interactions [13], [14]. The commonly utilized physicochemical characteristic of membrane rafts, i.e., their insolubility in detergents at low temperature, allows for the isolation of detergent-resistant membrane (DRM) fractions and thus the biochemical characterization of membrane rafts.

The localization of some HSPGs to the membrane rafts has been suggested in previous studies [15], [16], however those studies employed mostly the overexpression of chimeric proteins. Membrane and cytoplasmic domains of syndecan-1 were fused to ectodomain of Fc receptor [15] and had unknown physiological properties. Tkachenko and Simons, using similar syndecan-4 and Fc chimera, showed previously that redistribution of Fc-syndecan-4 complexes into raft membrane domains can be induced by clustering with IgG that specifically recognized the Fc receptor [16]. HSPGs are strategically located at the cell surface and are used for intercepting and regulating biological signals coming into cells, suggesting that localization of HSPGs to the specific domains on the cell surface would play an important role in the regulation of signaling pathways. In this study, we isolated and biochemically characterized native HSPG species localized to membrane domains of rat parathyroid (PTr) cells, where metabolism of HSPG has been well established [17]–[25].

## Results

### Isolation and characterization of detergent-resistant membranes from a [^35^S]sulfate-labeled rat parathyroid cell line

**Figure 1:**
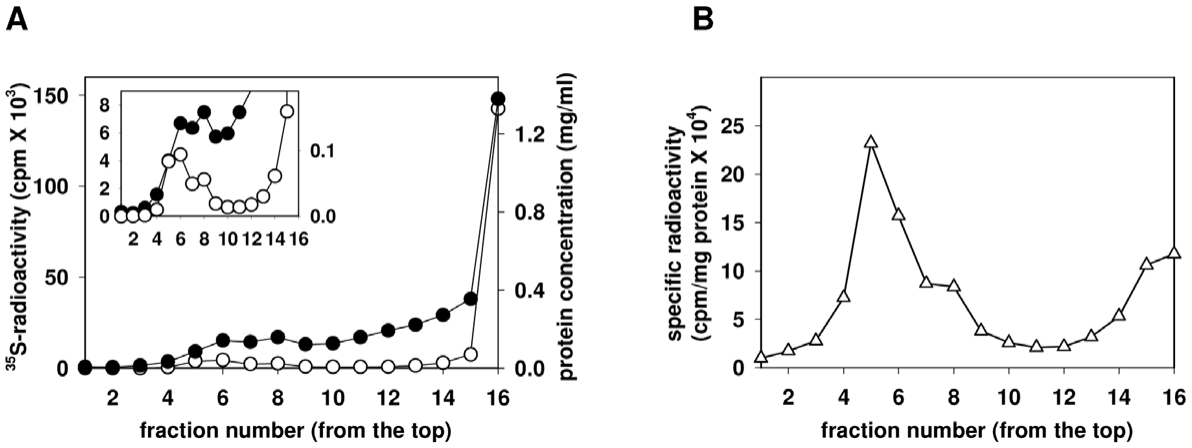
Sucrose-density gradient analysis of DRMs obtained from a [^35^S]sulfate labeled rat parathyroid cell line. Rat parathyroid cells (2×10^7^) were metabolically labeled with [^35^S]sulfate, and subjected to a preparation of DRMs as described in *Materials and Methods*. Fractions (200 µl each) were collected and counted for radioactivity after removal of free [^35^S]sulfate. A. Graph shows total radioactivity (○) and protein concentration (•) detected in each fraction. *Inset*, the same experimental data plotted in an expanded scale, emphasizing the presence of ^35^S-labeled material in low-density fractions. B. Graphic representation of specific radioactivity calculated for each fraction. The specific activity was expressed as total radioactivity per amount of the total protein material. DRM fractions were characterized by high specific activity suggesting enrichment with [^35^S]sulfate labeled molecules.

**Figure 2:**
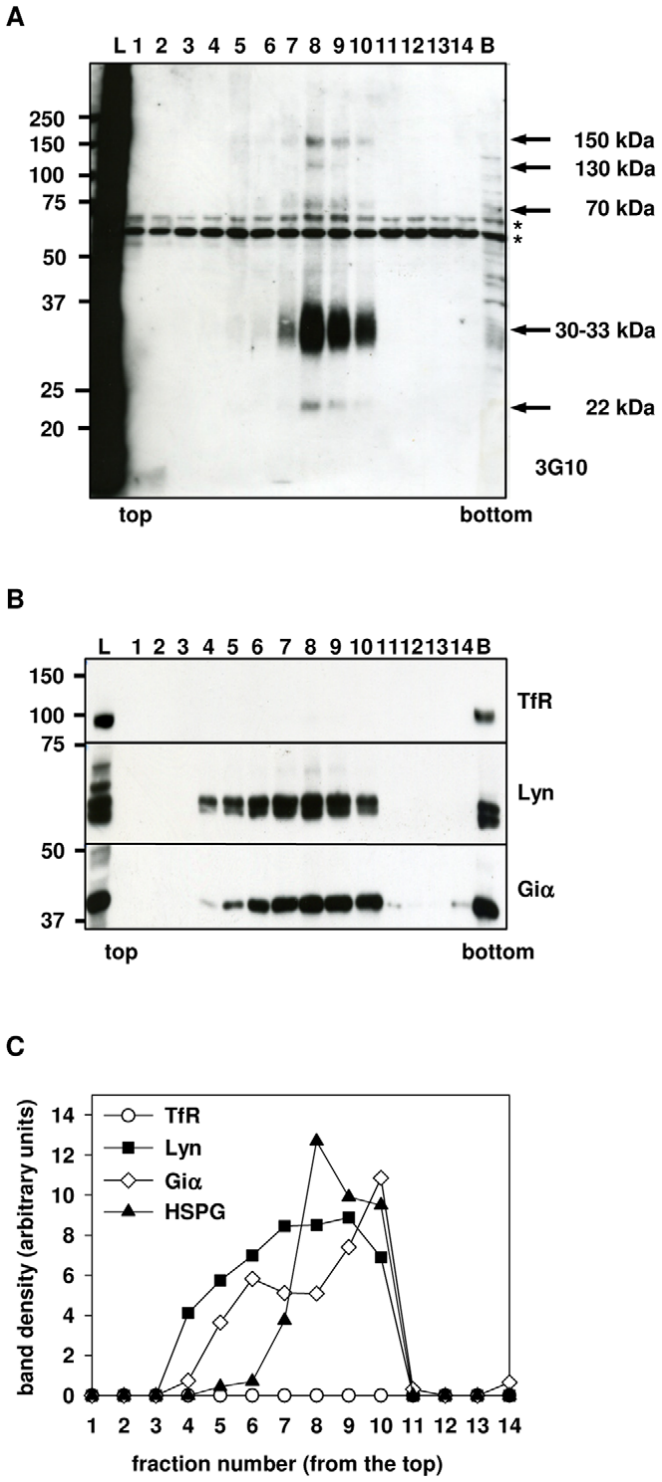
Western blotting analysis of DRM fractions isolated from a rat parathyroid cell line. DRMs were prepared from confluent PTr cells as described in *Materials and Methods*. Collected fractions, were concentrated, treated with heparitinase I and subjected to SDS-PAGE and WB analysis. A. Staining with anti-ΔHS (3G10) antibodies confirmed the presence of HSPGs in low-density fractions. Equal volumes (13 µl) of each fraction were analyzed. Fractions 13 and 14, bottom fraction (pooled fractions 15 and 16, B) and lysate (L) were diluted 16, 62 and 56 times, respectively, prior to the analysis. Bands marked with (*) represent non-specific staining due to the presence of BSA at high concentration. B. Staining with antibodies against DRM markers, Lyn and Giα defined the low-density fractions as DRMs. Equal volumes (33 µl) of each fraction were used for analysis. Fractions 13 through 14, bottom fraction (pooled fractions 15 and 16, B) and lysate (L) were diluted 18, 72 and 64 times, respectively, prior the analysis due to high protein content. Staining for transferrin receptor (TfR) was used as a control for the successful preparation. C. Graphic representation of the distribution of TfR, Lyn, Giα and HSPGs in fractions obtained from sucrose-density gradient ultracentrifugation. Density of bands detected in WB analysis (A and C) was measured and expressed as arbitrary units. TfR (○); Lyn (▪); Giα (◊) and HSPG (▴).

We have examined whether HSPGs in PTr cells are localized to membrane rafts. DRMs were isolated as described in the method section. Briefly, PTr cells at approximately 70% confluency were metabolically labeled with [^35^S]sulfate for 24 h, lysed with 1% Triton X-100 and subjected to sucrose-density gradient ultracentrifugation at 4°C. Each fraction was then assayed for radioactivity and protein concentration. The preparation showed a typical protein distribution pattern, in which most of the proteins became solubilized and recovered in high-density fractions at the bottom (Fig. 1A). Low-density fractions showed low protein content, with enrichment of proteins specific for DRMs, e.g., Lyn and Giα (Fig. 2B), confirming successful preparation of DRMs. The peak of [^35^S]sulfate-labeled material associated with macromolecules was detected in low-density fractions (Fig. 1A, *inset*). Specific radioactivity ([^35^S]sulfate/mg protein) calculated for each fraction showed higher values in low-density fractions compared to high-density ones (Fig. 1B), implying the enrichment of low-density fraction with specific proteoglycan species. Our previous analyses of PTr proteoglycans using metabolic radiolabeling showed that PTr cells produced almost exclusively HSPGs (>95%) [17], suggesting that [^35^S]sulfate-labeled material detected in DRM fractions represented HSPG species. In addition, studies done in our laboratory showed that in PTr approximately 25% of the ^35^S-labeled, cell-associated HSPGs were present on the cell surface while the rest resided intracellularly under similar culture conditions [18], [19]. Thus, under our culture conditions, the total HSPGs detected in DRMs likely represented at least 15% of the HSPGs residing on the cell surface, based on their trypsin accessibility. Quantitative analysis showed that 60–70% of [^35^S]sulfate-labeled material detected in low-density fractions (data not shown) could be removed by the trypsin treatment described in the Materials and Method section, suggesting that the majority of DRM-associated proteoglycans resided in trypsin-accessible compartment and thus originated from the cell surface, which is in a good agreement with the detailed report by Takeuchi et al. [18]. Western blotting (WB) analysis using antibodies recognizing HSPG was employed to confirm the identity of [^35^S]sulfate-labeled macromolecules as HSPG species. The unlabeled PTr cells were lysed and subjected to the same sucrose-density gradient ultracentrifugation procedure. Collected fractions were assayed for protein concentration and NADase activity (data not shown) and concentrated. Fractions were then treated with heparitinase I and anti-ΔHS antibodies (3G10) were used to detect HSPG. Polyacrylamide gel electrophoretic analysis of each fraction, revealed several bands with apparent MW of 150, 130, 70 kDa, 30–33 kDa and 22 kDa representing core proteins with HS stabs and thus presence of HSPGs (Fig. 2A). Treatment with chondroitinase ABC did not influence migration of detected bands (data not shown), confirming the absence of chondroitin sulfate chains as reported previously [17]. The 3G10-positive bands were detected in low-density fractions that found to be also positive for membrane raft markers, Lyn kinase and Giα (Fig. 2B, C), identifying the low-density fractions as membrane rafts-derived DRMs. Nonetheless, transferrin receptor (TfR) that is known to be excluded from membrane rafts was found only in a bottom fraction (Fig. 2B), suggesting that HSPG detected in DRMs fraction, specifically associates with membrane rafts.

### Disruption of detergent-resistant membranes using methyl-β-cyclodextrin (MβCD)

**Figure 3:**
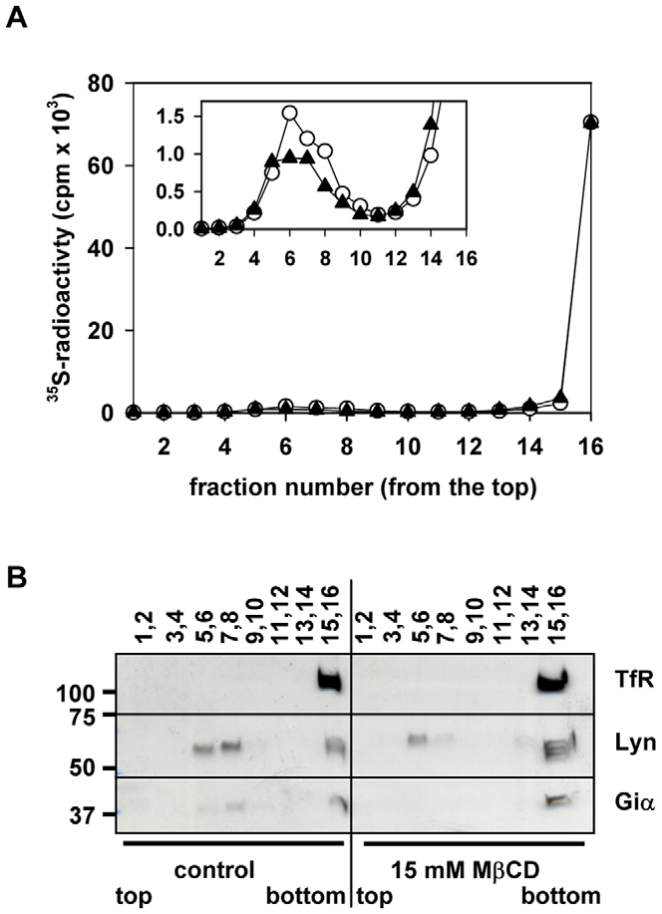
Sucrose-density gradient centrifugation analysis of DRM fractions obtained after MβCD treatment. Metabolically labeled PTr cells were incubated in the presence or absence of 10 mM MβCD or 15 mM MβCD for 1 h, followed by the isolation of DRMs as described in *Materials and Methods*. Fractions (200 µl each) were collected and either counted for ^35^S-radioactvity after removal of free [^35^S]sulfate or subjected to SDS-PAGE and immunoblotting with anti-DRM-specific marker antibodies. A. Radioactivity of DRM fraction decreased after MβCD treatment; (○) control, cells incubated in serum-free medium with 0.1% BSA; (▴) cells treated with serum-free medium, containing 10 mM MβCD, 0.1% BSA. *Inset*, the same experimental data showed in an expanded scale to allow the comparison of changes in radioactivity in low-density fractions, before and after treatment with MβCD. The release of HSPGs was calculated based on the total radioactivity of DRM fractions isolated from MβCD-treated cells, and expressed as % of the total activity of the DRM fractions obtained from control cells. B. Staining with antibodies against DRM markers, Lyn and Giα. Equal volumes of each fraction were used for WB analysis. The recovery of transferrin receptor (TfR) in bottom fractions was used as a control for the successful preparation.

The specific association of HSPGs with membrane rafts was further examined. Metabolically [^35^S]sulfate-labeled PTr cells were treated with a cholesterol-complexing agent, MβCD, in order to deplete cholesterol from DRMs and disrupt their integrity [26], followed by sucrose-density gradient ultracentrifugation. Analysis of membrane preparation showed 30–40% reduction (variable among experiments) in the total radioactivity of DRM fractions isolated after MβCD treatment when compared to the ones from the untreated control (Fig. 3A). This implies that only part of HSPGs was associated with cholesterol-rich DRMs. Partial resolution of this fraction by MβCD might be due to the limitation of MβCD treatment or association of HSPGs with a subpopulation of membrane rafts which could not be completely disrupted by MβCD. The first possibility, however, was not likely, since the further increase in MβCD concentration (up to 15 mM) did not significantly change the amount of HSPGs detected in DRM fractions (data not shown), but caused complete dissociation of Giα from DRMs (Fig. 3B). Lyn kinase was still detectable in DRMs, even after treatment with 15 mM MβCD (Fig. 3B), but Lyn bears two acyl chains and is likely to be more tightly associated with DRMs than Giα that has only one acyl chain [27]. Membrane rafts are heterogeneous structures in regards to their composition, size and turnover rate [28]. Furthermore, degree of cholesterol depletion may vary between cell types even if comparable MβCD concentrations are employed [29], so one may consider that in PTr cells, membrane rafts enriched with HSPG could be only partially susceptible to MβCD treatment.

### Identification of HSPGs present in DRMs isolated from a rat parathyroid cell line

**Figure 4:**
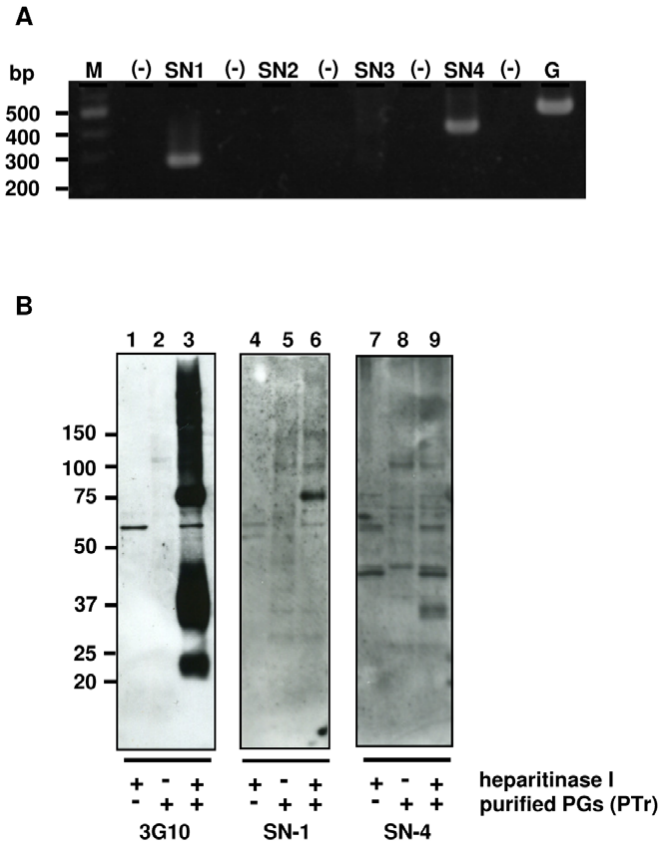
Identification of HSPGs expressed by a rat parathyroid cell line. A. RT-PCR analysis of PTr cells using syndecan-specific primers (see *Materials and Methods* for details). Total RNA was isolated from confluent cells and subjected to RT-PCR analysis. Amplified products were run on 2% agarose gel, stained with ethidium bromide and photographed under UV transilluminator. Lanes: M – 100 bp marker; SN1 – amplification with syndecan-1 specific primers; SN2 – amplification with syndecan-2 specific primers; SN3 – amplification with syndecan-3 specific primers; SN4 – amplification with syndecan-4 specific primers; G – amplification with GAPHD specific primers; (-) – negative controls containing no cDNA. B. Identification of HSPGs present in DRM fractions using WB analysis. Proteoglycans were isolated from confluent rat parathyroid cells and partially purified using Q-Sepharose anion-exchange chromatography. A proteoglycan-enriched fraction was incubated in the presence or absence of heparitinase I, subjected to SDS-PAGE and immunoblotted with anti-syndecan-1, anti-syndecan-4 or anti-ΔHS (3G10) antibodies. Lanes: 1, 4 and 7 represent the heparitinase I only; 2, 5 and 8 correspond to the control samples, incubated without heparitinase I; 3, 6, 8 correspond to the heparitinase-treated samples.

Analysis of an apparent MW of core proteins detected in WB analysis suggested the detected HSPGs to be members of syndecan and/or glypican family. To determine what types of HSPG are expressed by PTr cells, the total RNA was isolated from cultured cells and subjected to reverse transcriptase polymerase chain reaction (RT-PCR) analysis using sets of primers specific for syndecan-1, -2, -3 and -4, respectively (see Materials and Methods). Analysis of PCR products showed syndecan-1 and -4 to be the only members of syndecan family expressed by PTr cells (Fig. 4A). PTr cells were found also to express GPI-anchored HSPG species. RT-PCR revealed the expression of glypican-1 and glypican-4 (data not shown) among six glypicans analyzed (glypican-1 through glypican-6), indicating them as potential candidates for lipid raft-associated HSPGs [30].

WB analysis using anti-syndecan antibodies was done to identify the core proteins detected in low-density fractions. Due to low protein contents in DRMs isolated from PTr cells, attempts to stain directly DRM fractions with syndecan specific antibodies were not successful. Therefore, an indirect procedure was undertaken. HSPGs were isolated using an anion-exchange chromatography, digested with heparitinase I and subjected to WB analysis using various anti-syndecan antibodies [31], [32]. The results were compared with the pattern of staining with anti-ΔHS antibodies (3G10). Staining with anti-syndecan antibodies identified bands with apparent MW of 30–33 and 70 kDa as syndecan-4 and syndecan-1, respectively (Fig. 4B), suggesting both to be specifically associated with membrane rafts of PTr cells. In addition, we also analyzed targeting of syndecans to membrane rafts using PTr cells overexpressing syndecan-1 and syndecan-4 tagged with green fluorescent protein (GFP). DRM fractions isolated from either transfectants showed the presence of GFP-tagged proteins, reactive to both 3G10 and anti-syndecans antibodies (Fig. S1B and S1C), confirming the targeting of both syndecans to membrane rafts. Targeting of both syndecans to membrane rafts was not GFP-dependent. GFP expressed alone was found mostly in the bottom fractions (Fig. S1A). In RT-PCR analysis PTr cells showed also expression of glypican-1 and glypican-4 (data not shown), however attempts to detect glypicans in DRM fractions were not successful due to the lack of good detection tools, i.e., specific antibodies.

### Characterization of DRMs containing HSPGs

**Figure 5:**
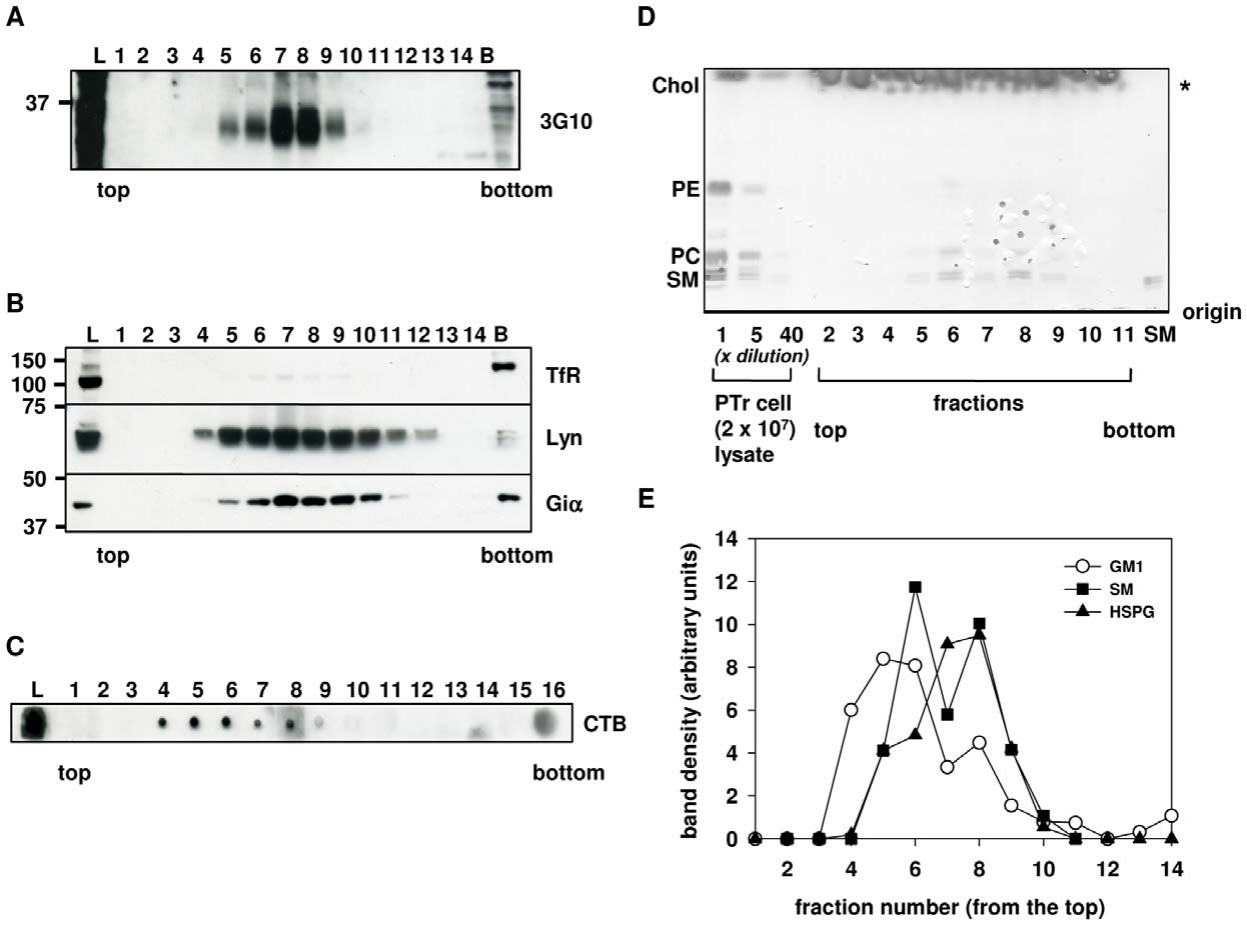
Characterization of DRM fractions isolated from a parathyroid cell line. Confluent PTr cells were subjected to DRMs preparation as described in *Materials and Methods*. Concentrated fractions were analyzed for presence of HSPGs by WB. They were also examined for the presence of ganglioside GM1 by the binding of cholera toxin B (CTB) subunit and sphingomyelin (SM) by HPTLC analysis. A. Staining with 3G10 antibodies confirmed the presence of HSPGs in low-density fractions. Equal volumes (13 µl) of each fraction were used for analysis. Fractions 13 and 14, bottom fraction (pooled fractions 15 and 16, B) and lysate (L) were diluted 16, 62 and 56 times, respectively, prior to the analysis. B. DRM markers, Lyn and Giα showed a broad distribution, but partially overlapped with low-density fractions containing HSPGs. Equal volumes (33 µl) of each fraction were used for analysis. Fractions 13 through 14, bottom fraction (pooled fractions 15 and 16, B) and lysate (L) were diluted 18, 72 and 64 times, respectively, prior the analysis due to high protein content. Successful preparation was confirmed by immunostaining for TfR, which was found mainly in the bottom fractions. C. An equal aliquot (2 µl) of each fraction was dot-blotted onto PVDF membrane and stained with HRP-conjugated CTB subunit. Numbers correspond to the fraction number; L, original lysate before sucrose-density gradient ultracentrifugation. D. Lipids extracted from sucrose density gradient fractions (fractions 2–11) were developed on the HPTLC plate and stained with 3% cupric acetate and 8% phosphoric acid. Chol, cholesterol; PE, phosphoethanolamine; PC, phosphocholine; SM, sphingomyelin. Non-specific staining due to the presence of traces of detergent in samples is marked with (*). E. Graphic representation of distribution of HSPGs, ganglioside GM1 and SM in DRM fractions. Low-density fractions containing HSPGs showed enrichment with SM. Density of bands detected in WB and HPTLC was measured with ImageJ and expressed in arbitrary units. Graph shows distribution of ganglioside GM1 (○); HSPGs (▴) and SM (▪).

Each fraction of sucrose gradient was examined for the presence of HSPGs, TfR, Lyn, Giα as well as lipid content to characterize the HSPG-containing DRMs further. Sucrose-density gradient fractions from unlabeled PTr cells were prepared as outlined in Materials and Methods, and analyzed again by WB with anti-ΔHS antibodies (3G10). The 3G10-positive material was distributed throughout fractions 5–9, with the most intense staining for fractions 7 and 8 (Fig. 5A). Both Lyn and Giα showed different distribution when compared to HSPGs (Fig. 5B), Lyn was distributed throughout fractions 4–12, while Giα was detected throughout fractions 5–11 suggesting the heterogeneity of recovered DRMs. Kiyokawa and colleagues reported different distribution and functional diversity of the membrane rafts enriched with SM and ganglioside GM1 [10]. Therefore, HSPG-enriched DRMs were also analyzed for lipid content as described in Materials and Methods. HSPG-enriched DRM fractions, found to be also enriched with SM, as confirmed by high-perfomance thin-layer chromatography (HPTLC) analysis (Fig. 5D). The distribution of ganglioside GM1was examined by staining with the cholera toxin B (CTB) subunit. CTB staining showed that, in contrast to HSPG, the material derived from GM1-enriched microdomains tended to distribute in fractions of lower density (Fig. 5C). Our previous experiments showed that these GM1-enriched DRMs were characterized by high cholesterol content. Cholesterol distribution overlapped more closely with the distribution of CTB-positive material (Podyma-Inoue and Yanagishita, unpublished observations).

Previous reports suggested that in myeloma cells, HS chains regulate the targeting of syndecan-1 to uropod, a specialized domain on the cell surface [33]. Thus, it is possible that the presence of HS chains influence the recruitment of syndecans to the lipid rafts in PTr cells. This hypothesis was examined by removing HS chains from the cell surface. The cells were treated with heparitinase I for 1 hour before the lysis with 1% Triton X-100, and subjected to sucrose-density gradient ultracentrifugation. The WB analysis of obtained fractions using 3G10 antibodies, showed that core proteins of HSPGs were retained in DRMs (Fig. S2), suggesting that the intact HS chains are not required for HSPGs recruitment to the membrane rafts in PTr cell.

## Discussion

Many studies have shown the importance of HSPGs in ligand-receptor [34] and cell-extracellular matrix interactions [35]. The localization of HSPGs on the cell surface and mechanisms by which HSPGs are maintained within the specific cell surface structures, critically affect biological functions of HSPGs. Thus, the approach taken in the present study aimed at the biochemical characterization of plasma membrane domains that are involved in targeting of ligands to specific subcellular compartments, focusing on the isolation and characterization of HSPG species that specifically localize to membrane domains of PTr cells.

Metabolic labeling experiments in combination with sucrose-density gradient ultracentrifugation indicated the presence of HSPGs in DRM fractions (Fig. 1A). Recent reports suggested the presence of HSPG in membrane rafts however those attempts were based on analysis of overexpressed chimeric protein consisting of syndecan and Fc amino acid sequences [15], [16]. Our report demonstrated the physical presence of the endogenously expressed HSPGs, both syndecan-1 and syndecan-4 as well as yet unidentified HSPGs, in the raft microdomains of the cell membrane (Fig. 2A and 4B). The amount of HSPGs associated with DRM fractions varied between 4 to 10% of the total cellular HSPGs depending on preparations (Podyma-Inoue and Yanagishita, unpublished observations), likely reflecting the dynamic state of membrane rafts [18]. The amount of ^35^S-labeled HSPGs associated with DRM fractions corresponded to 15% of the cell surface HSPGs in the rafts as estimated by metabolic radiolabeling experiment and accessibility to trypsin (data not shown) and was in good agreement with amount other protein abundant in membrane rafts, e.g., Lyn or Giα (13–31% and 8–14%, respectively, as estimated by WB analysis; Fig. 2B).

Reduction of cholesterol content by MβCD only partially removed HSPGs from plasma membrane rafts (Fig. 3A). Increasing the concentration of MβCD beyond 15 mM did not further reduce the proportion of HSPGs recovered in DRM fraction (data not shown), implying that HSPG-containing membrane rafts are only partially susceptible to MβCD treatment. This hypothesis is supported by the heterogeneity of membrane rafts [28] as well as our analysis of the lipid composition of recovered DRM fractions. HSPG-DRMs were recovered together with SM-enriched membranes (Fig. 5E) and thus, could be potentially less susceptible to MβCD treatment.

WB analysis revealed association of several HSPG species with DRMs (Fig. 2). The estimated MWs of detected bands (150 kDa, 130 kDa) were in good correlation with a previous report regarding characterization of proteoglycans synthesized by PTr cells [17]. The presence of other HSPGs (70 and 30–33 kDa) was likely due to improved detection techniques or different sample preparation. These two DRM-associated HSPGs (70 and 30–33 kDa) could be identified as syndecan-1 and syndecan-4, respectively (Fig. 4B) whereas the identity of other HSPGs remains to be determined. However, based on our previous report, these HSPGs with apparent MWs of 150 kDa and 130 kDa are unique to PTr cells [17].

PTr cells express also two types of GPI-anchored proteoglycans, glypican-1 and glypican-4 (Podyma-Inoue and Yanagishita, unpublished data). However, in the present study, these two glypicans could not been identified. Attempts to detect the endogenous proteins were not successful probably due to the low levels of expression and the lack of an appropriate antibodies or their minimal association with membrane rafts. In fact, although many of GPI-anchored proteins are recruited to membrane rafts the amino acid sequence of the core protein itself appears to be an important factor for membrane microdomain localization, making the presence of GPI-anchor alone insufficient for recruitment to membrane rafts [36].

Kiyokawa and collaborators have shown that in Jurkat T cells, SM-enriched membranes distribute differently from GM1-enriched membrane microdomains and are functionally distinct [10]. SM- and GM1-rich domains seem to serve as platforms for different cellular signaling; i.e., SM-rich domains play a role in signaling events dependent on G protein-coupled receptors through intracellular calcium ion mobilization and ERK 1/2 phosphorylation, while GM1-rich domains mediate the signaling related to T cell receptor via Src-family kinase activation pathways [10]. In PTr cell line, low-density fractions enriched with HSPGs were characterized by high SM content (Fig. 5D) and were found to only partially overlap with the fractions enriched with GM1 (Fig. 5C and E) and cholesterol (data not shown). The important role of HSPGs as high affinity receptors for FGF in PTr cell line has been demonstrated [20] suggesting the possibility of the putative involvement of HSPG-enriched rafts in FGF signaling. This hypothesis is supported by the recent reports that suggest importance of syndecan-4 in the activation of ERK 1/2 via FGF/FGFR signaling [37].

The mechanism of recruitment of HSPGs to membrane rafts remains elusive. The role of both protein core and HS chains, have been implicated, but no clear answer has been revealed. Detailed biochemical investigation of HSPGs present on the cell surface combined with studies on the internalization of HSPGs will help to define the molecular nature of HSPG-ligand interactions. Further proteomic characterization of HSPG-enriched membrane domains might identify the proteins that interact directly or indirectly with HSPGs in the membrane rafts and determine signaling/endocytic complexes.

## Materials and Methods

### Materials

A reagent for total RNA isolation (RNA STAT-60™) was purchased from TEL-TEST, Inc. (Friendswood, TX). [^35^S]Sulfate (25 mCi) was obtained from PerkinElmer New England Nuclear (Waltham, MA). Rabbit anti-Lyn (Lyn 44) polyclonal antibodies were purchased from Santa Cruz Biotechnology (Santa Cruz, CA). Rabbit anti-α subunit of transducin Gi1 and Gi2 (Giα) polyclonal antibodies were from Du Pont (Boston, MA). Biotinylated mouse anti-ΔHS (3G10) antibodies, recognizing HS neo-epitope, generated by the digestion with heparitinase I from *Flavobacterium heparinum* and heparitinase I (*Flavobacterium heparinum*) were purchased from Seikagaku Corporation, (Tokyo, Japan). Anti-syndecans antibodies were generously provided by Dr. Minoru Okayama (Kyoto Sangyo University, Kyoto, Japan) and Dr. Pyong W. Park (Children's Hospital Boston, Boston, MA). Monoclonal antibodies JL-8 recognizing green fluorescent protein (GFP) were purchased from Clontech (Mountain View, CA). Other reagents used were of the highest grades commercially available.

### Metabolic radiolabeling of cell cultures

PTr cells [38] were metabolically radiolabeled as reported previously [17]. Briefly, cells at approximately 70% confluency were incubated for 24 h at 37°C under 95% air/5% CO_2_ in Minimum Essential Eagle's Medium/Coon's modified Ham's F-12 (MEM/F-12 in 1∶1 ratio) (Sigma, St. Louis, MO) supplemented with 5% calf serum (Gibco, Auckland, N.Z.) in the presence of [^35^S]sulfate at the concentration of 50 µCi/ml. After labeling, the cells were washed five times with non radioactive medium to remove the excess of free [^35^S]sulfate. The cells were then scraped off with the rubber policeman in ice-cold Mg^+2^- and Ca^+2^-free phosphate-buffered saline (PBS) and subjected to the preparation of detergent-resistant membranes (DRMs).

Removal of cholesterol from the plasma membrane was achieved by treatment of the cells with methyl-β-cyclodextrin (cholesterol-complexing agent). In brief, after metabolic radiolabeling, confluent cells were washed twice with prewarmed serum-free medium and incubated with 10 mM MβCD (Sigma, St. Louis, MO) for 1 h, at 37°C. Cells were then washed twice with ice-cold PBS, scraped off and subjected to preparation of DRMs.

For the quantification of proteoglycans residing in trypsin accessible compartments, the cells were washed twice with serum-free medium for 15 min at 37°C, followed by treatment with trypsin (50 µg/ml) in serum-free medium for 2 min at 37°C. Released material was collected and cells were subjected to additional treatment with trypsin (50 µg/ml) in serum-free medium for additional 15 min at 37°C. Proteoglycans present in both released materials, were then isolated using Sephadex G-50 as described in later section.

### Preparation of DRMs

Metabolically radiolabeled or unlabeled PTr cells (2×10^7^) were scraped off and collected by centrifugation, at 4°C. The cells were then lysed in 200 µl of 50 mM Tris-HCl, 25 mM KCl buffer, pH 6.8, containing 1% Triton X-100 for 30 min, at 4°C, with vigorous vortexing every 5 min. Lysate was adjusted to 40% sucrose by addition of an equal volume of 80% sucrose in 50 mM Tris-HCl, 25 mM KCl buffer, pH 6.8. Lysate was then placed into 5 ml centrifuge tube and overlaid with 2 ml of 30% sucrose and 1 ml of 5% sucrose in 50 mM Tris-HCl, 25 mM KCl, pH 6.8 to form discontinuous gradient. The material was centrifuged at 45,000 rpm at 4°C for 16–20 h in RPS65-TA rotor (Hitachi Koki, Tokyo, Japan). Sixteen fractions (200 µl each) were collected from the top of the gradient. Each fraction was assayed for protein content using BCA Protein Assay Kit (Pierce, Rockford, IL) and NAD+ glycohydrolysis [39] to confirm the successful preparation. Fractions were then either subjected to isolation of proteoglycans or collection of DRMs by ultracentrifugation at 70,000 rpm and 4°C for 30 min in TLA 100.2 rotor (Beckmann, Fullerton, CA) followed by WB analysis. To identify the proteoglycan core proteins, samples were digested with heparitinase I prior to WB analysis. Enzymatic digestion was carried out at 37°C for 1 h, in 0.1 M Tris-acetate, 10 mM calcium acetate buffer, pH 7.3, in the presence of 2 mU of enzyme.

In order to examine the influence of the removal of HS chains on the integrity of membrane rafts the confluent cells were washed three times with PBS and incubated with heparitinase I (0.1 U/ml) in PBS in, 10 mM calcium acetate for 1 h, at 37°C prior the preparation of DRMs.

### Isolation of [^35^S]sulfate-labeled macromolecules

Fractions obtained from sucrose-density-gradient ultracentrifugation were made up to 4 M in guanidine HCl and applied onto a Sephadex G-50 (8 ml bed volume, GE Healthcare, Buckinghamshire, UK) column equilibrated with 8 M urea, 0.2 M NaCl, 0.05 M sodium acetate, pH 6.0 containing 0.5% Triton X-100. Excluded volume fractions were collected and radioactivity was measured with OptiPhase “HighSafe” 3 scintillation cocktail (Wallac, Turku, Finland) using a Beckmann liquid scintillation counter (Fullerton, CA). The results were expressed as a total radioactivity present in each fraction.

### Analysis of expression of HSPG mRNAs using RT-PCR

Rat parathyroid (PTr) cells were maintained in MEM/F-12 (1∶1) supplemented with 5% calf serum. Total RNA was isolated using RNA STAT-60™ reagent according the protocol supplied by the manufacturer. cDNA was synthesized from 2 µg of total RNA using Superscript™ II RNase H^-^ Reverse Transcriptase (Invitrogen, Carlsbad, CA) under the conditions suggested by the manufacturer. Expression of each syndecan family member was determined using the set of specific primers as listed below: syndecan-1 (SN-1) forward 5′-TTGTCACCGCAAATGTGCCTCC-3′, reverse 5′-AGTGAAGTCAGGCTCTGCTTCC-3′, syndecan-2 (SN-2) forward 5′- ATATGCAGCGTGCGTGGATCC-3′, reverse 5′-TCGTCTTTCTTCCGCATGCGG-3′, syndecan-3 (SN-3) forward 5′-TGGATCTTGAGGGCTCAGGGG-3′, reverse 5′- TGCTGTGGCAGGTGCTGTGG-3′, syndecan-4 (SN-4) forward 5′-GAGTCGATTCGAGAGACTGAGG-3′, reverse 5′-AAAATGTTGCTGCCCTGGG-3′, GAPDH forward 5′-ACCACAGTCCATGCCATCAC-3′, reverse 5′-TCCACCACCCTGTTGCTGTA-3′. Amplification was carried out in Perkin Elmer GeneAmp PCR System 9600 (Perkin Elmer, Norwalk, CA) in 30 cycles of three-step reaction (denaturation at 94°C for 30 s, annealing at 64°C for 30 s, extension at 72°C for 60 s). PCR products were analyzed by 2% agarose gel electrophoresis in TAE buffer. DNA bands were visualized with ethidium bromide and photographed under UV transillumination. The nucleotide sequence data used for primer design are registered in GenBank under the following accession numbers: rat syndecan-1 mRNA, NM_013026; rat syndecan-2 mRNA, NM_013082; rat syndecan-3 mRNA, NM_053893; rat syndecan-4 mRNA, NM_012649; rat GAPDH mRNA, NM_017008.

### Purification of proteoglycans from PTr cells

Confluent PTr cells (2×10^7^) were washed twice with PBS at 4°C and subjected to proteoglycan purification as described elsewhere [40]. Briefly, cells were extracted overnight with 10 ml of 4 M guanidine HCl, 2% Triton X-100, at 4°C. The buffer was exchanged using Sephadex-G50 column (8 ml bed volume) equilibrated with 8 M urea, 0.2 M NaCl, 0.2 M sodium acetate, pH 6.8, containing 0.5% Triton X-100. Material recovered from the excluded volume fraction of Sephadex-G50 chromatography, was then mixed with Q-Sepharose slurry, in the same urea buffer, and allowed to bind for 2 h, at room temperature. The slurry was packed into a column and extensively washed, the bound macromolecules were eluted with 4 M guanidine HCl, 0.5% Triton X-100. Obtained material was then dialyzed against PBS, treated with heparitinase I and used in WB analysis.

Purification of proteoglycans from fractions obtained in sucrose-density gradient ultracentrifugation was done in similar way. Briefly, [^35^S]sulfate-labeled molecules were present in DRMs and bottom fractions were isolated using Sephadex-G50 as described in earlier section. DRM- and bottom fraction-associated macromolecules were then pooled respectively and subjected to purification of proteoglycans using Q-Sepharose.

### Establishment of PTr cells expressing syndecan-1-GFP and syndecan-4-GFP

PTr transfectants were established using Trap-In gene expression system developed and described by Shinomura and collaborators [41]. Briefly, PTr cells were infected with the retroviral vector prvPtrap, containing hygromycin-resistance and β-galactosidase genes, (GenBank accession number AB375112). Cells were then cultured in hygromycin selective medium followed by isolation of Trap-In host cell line (PTrβ). This cell line was characterized by high expression of β-galactosidase reporter gene and was used for expression of syndecan-GFP fusion proteins. Expression vectors were prepared as followed: cDNA for GFP was prepared from pEGFP-N3 vector (Clontech, Mountain View, CA) by digestion with BamHI and NotI and inserted into pInSRT vector (GenBank accession number AB375113). Next, full-length rat syndecan-1 or syndecan-4 genes were amplified with gene-specific primers, respectively and were ligated into pInSRT-GFP vector giving rise to pInSRT-SN1-GFP and pInSRT-SN4-GFP, respectively. PTrβ cells were co-electroporated with FLP recombinase expression vector (pOG44, Invitrogen, Carlsbad, CA) and pInSRT-SN1-GFP, pInSRT-SN4-GFP or pInSRT- GFP, respectively followed by selection with puromycin (3 µg/ml) for 3 weeks. Puromycin-resistant cells were pooled, grown and used for preparation of DRMs as described in the previous section.

### Autoradiography/SDS-PAGE and Western blotting analysis

Proteins were separated on 11–14% polyacrylamide gels (Dai-ichi Chemicals, Tokyo, Japan) according to the method of Laemmli [42] and transferred onto a polyvinylidene (PVDF) membrane (Millipore, Bedford, MA) using the wet-transfer system [43]. The membranes were either stained with colloidal gold (Bio-Rad Laboratories, Hercules, CA) or subjected to WB procedure. In the latter case, membranes were blocked with 1% bovine serum albumin (BSA) in PBS, 0.01% Tween 20 at room temperature for 40 min and incubated with specific antibodies (1∶500 dilution for both anti-syndecans, 1∶1000 dilution for 3G10 and 1∶1000 dilution for anti-GFP antibodies) in 1% BSA in PBS, 0.01% Tween 20, overnight at 4°C or room temperature for 3 h. After six washes of 5 min each, membranes were incubated for 1 h at room temperature with appropriate horseradish peroxidase-conjugated secondary antibodies. Following the extensive wash, immunoreactivity was determined using the ECL Detection System (GE Healthcare, Buckinghamshire, UK). Density of detected bands was determined using ImageJ Program [44].

### Analysis of lipid contents by high-performance thin-layer chromatography (HPTLC)

For determination of lipid content, DRM fractions (200 µl each) obtained from sucrose-density gradient ultracentrifugation (fraction 2 throughout 11) were collected by ultracentrifugation at 70,000 rpm and 4°C for 30 min in TLA 100.2 rotor (Beckmann, Fullerton, CA), rinsed twice with 1 ml PBS and extracted with 1 ml of methanol-chloroform (1∶1). Extracted lipids were dried under vacuum in centrifugal concentrator (TOMY SEIKO Co., Tokyo, Japan) and dissolved in 30 µl of chloroform-methanol (1∶1). Samples were then loaded onto a pre-coated high-performance TLC plate (20×10 cm), Silica Gel 60 (Merck, Darmstadt, Germany), developed in chloroform-methanol-water (65∶25∶4) and visualized with 3% cupric acetate and 8% phosphoric acid, followed by analysis with ImageJ Program [44].

## Supporting Information

Figure S1 **Characterization of DRM fractions isolated from a parathyroid cell line overexpressing syndecans tagged with GFP.** PTr cells stably expressing GFP, syndecan-1-GFP and syndecan-4-GFP fusion proteins were subjected to DRMs preparation as described in *Materials and Methods*. Concentrated fractions were analyzed for presence of HSPGs by Western blotting using 3G10 antibodies. After stripping off the 3G10 antibody, the same membranes were re-probed with anti-GFP antibodies followed by another cycle of stripping/re-probing with anti-syndecan-1 or anti-syndecan-4 antibodies, respectively. Equal volumes (13 µl) of each fraction were used for analysis. Fractions 13 and 14, bottom fraction (pooled fractions 15 and 16, B) and lysate (L) were diluted 16, 62 and 56 times, respectively, prior to the analysis. A. Analysis of control PTr cells expressing GFP only showed typical for PTr cells HSPG pattern (left panel). GFP was mostly found in the bottom fractions (right panel). B. Analysis of control PTr cells expressing syndecan-1-GFP fusion protein. Band detected in DRMs, representing 3G10-reactive material of an apparent molecular mass of 100 kDa (left panel) was also reactive to anti-GFP (middle panel) and anti-syndecan-1 antibodies (right panel), confirming the specific targeting of syndecan-1 to DRMs. C. Analysis of control PTr cells expressing syndecan-4-GFP fusion protein. Band of an apparent molecular mass of 58 kDa reacted with 3G10 (left panel), anti-GFP (middle panel) antibodies and anti-syndecan-4 antibodies (right panel). Materials likely representing partially digested syndecan-GFP fusion proteins, reactive to both anti-GFP and anti-syndecans antibodies are marked with (*).
(TIF)

Figure S2 **Analysis of role of HS chains for localization of HSPGs to membrane rafts.** Confluent PTr cells were treated with heparitinase I followed by preparation of DRMs as described in *Materials and Methods*. Concentrated fractions were analyzed for presence of HSPGs by Western blotting using 3G10 antibodies confirming the presence of HSPGs in DRMs even after heparitinase treatment. Equal volumes (13 µl) of each fraction were used for analysis. Fractions 13 and 14, bottom fraction (pooled fractions 15 and 16, B) and lysate (L) were diluted 16, 62 and 56 times, respectively, prior to the analysis. Bands marked with (*) represent non-specific staining due to the presence of BSA at high concentration.
(TIF)
