# Supplementary figures and images for: Syndecans Reside in Sphingomyelin-Enriched Low-Density Fractions of the Plasma Membrane Isolated from a Parathyroid Cell Line
Source: PLoS One. 2012 Mar 1;7(3):e32351. doi: 10.1371/journal.pone.0032351 (PMC3291576; doi:10.1371/journal.pone.0032351)

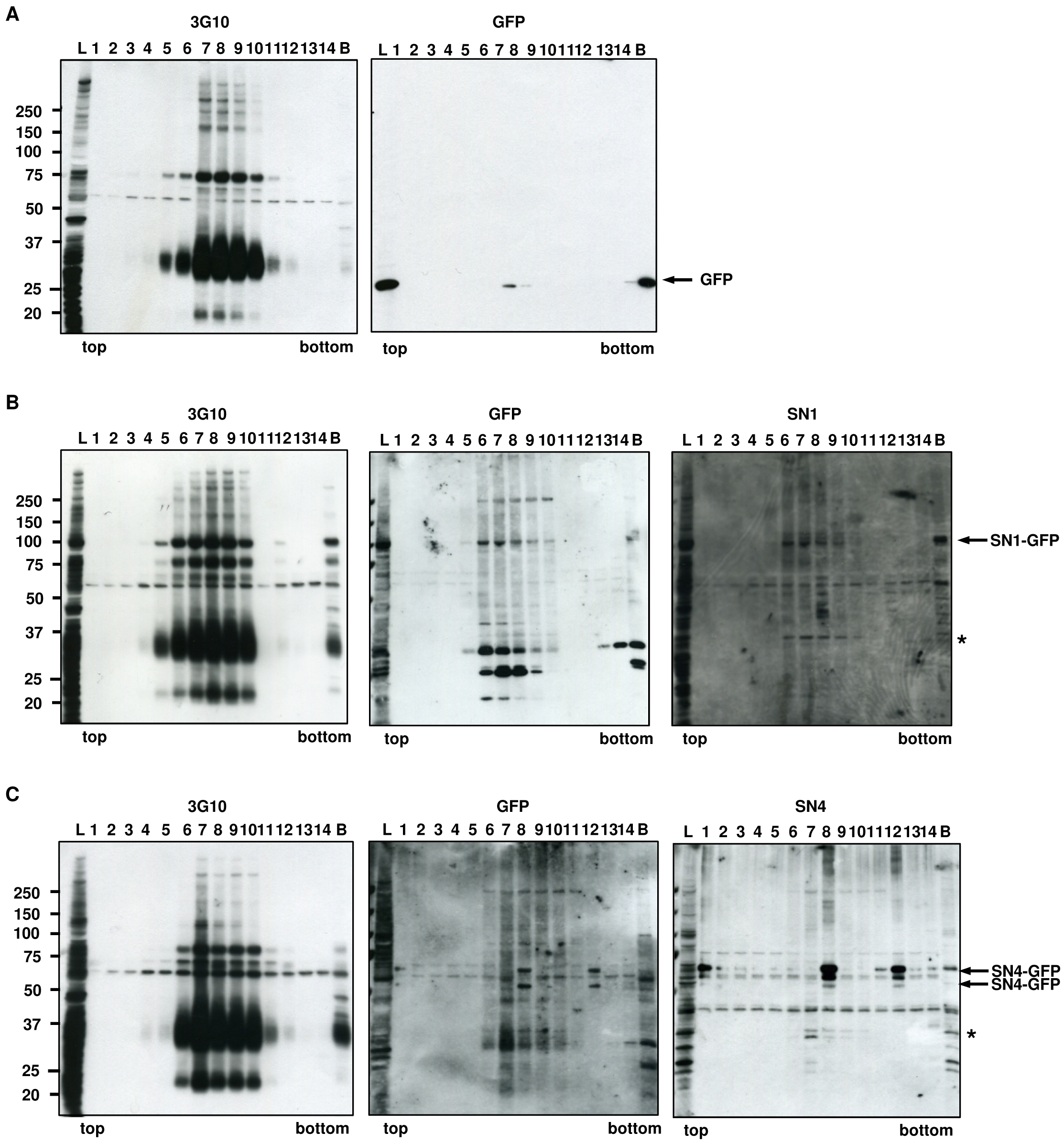

Supplement: Figure S1 — Characterization of DRM fractions isolated from a parathyroid cell line overexpressing syndecans tagged with GFP. PTr cells stably expressing GFP, syndecan-1-GFP and syndecan-4-GFP fusion proteins were subjected to DRMs preparation as described in Materials and Methods . Concentrated fractions were analyzed for presence of HSPGs by Western blotting using 3G10 antibodies. After stripping off the 3G10 antibody, the same membranes were re-probed with anti-GFP antibodies followed by another cycle of stripping/re-probing with anti-syndecan-1 or anti-syndecan-4 antibodies, respectively. Equal volumes (13 µl) of each fraction were used for analysis. Fractions 13 and 14, bottom fraction (pooled fractions 15 and 16, B) and lysate (L) were diluted 16, 62 and 56 times, respectively, prior to the analysis. A. Analysis of control PTr cells expressing GFP only showed typical for PTr cells HSPG pattern (left panel). GFP was mostly found in the bottom fractions (right panel). B. Analysis of control PTr cells expressing syndecan-1-GFP fusion protein. Band detected in DRMs, representing 3G10-reactive material of an apparent molecular mass of 100 kDa (left panel) was also reactive to anti-GFP (middle panel) and anti-syndecan-1 antibodies (right panel), confirming the specific targeting of syndecan-1 to DRMs. C. Analysis of control PTr cells expressing syndecan-4-GFP fusion protein. Band of an apparent molecular mass of 58 kDa reacted with 3G10 (left panel), anti-GFP (middle panel) antibodies and anti-syndecan-4 antibodies (right panel). Materials likely representing partially digested syndecan-GFP fusion proteins, reactive to both anti-GFP and anti-syndecans antibodies are marked with (*). (TIF) [file pone.0032351.s001.tif]

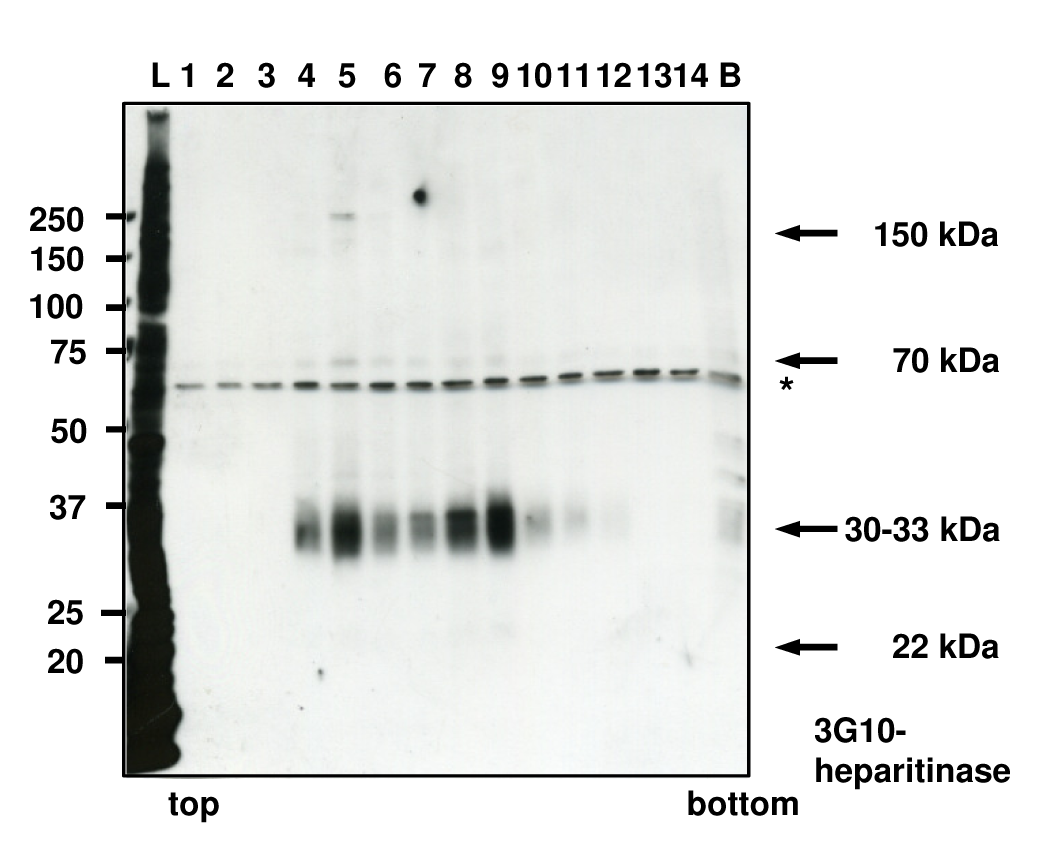

Supplement: Figure S2 — Analysis of role of HS chains for localization of HSPGs to membrane rafts. Confluent PTr cells were treated with heparitinase I followed by preparation of DRMs as described in Materials and Methods . Concentrated fractions were analyzed for presence of HSPGs by Western blotting using 3G10 antibodies confirming the presence of HSPGs in DRMs even after heparitinase treatment. Equal volumes (13 µl) of each fraction were used for analysis. Fractions 13 and 14, bottom fraction (pooled fractions 15 and 16, B) and lysate (L) were diluted 16, 62 and 56 times, respectively, prior to the analysis. Bands marked with (*) represent non-specific staining due to the presence of BSA at high concentration. (TIF) [file pone.0032351.s002.tif]
